# 783 Use of Multidisciplinary Collaboration to Establish a Standardized Endotracheal Tube Wiring Guideline

**DOI:** 10.1093/jbcr/irac012.334

**Published:** 2022-03-23

**Authors:** Lisa M Shostrand, Brett C Hartman, Madeline Zieger, Aimee Ealy, Diana M Meadors

**Affiliations:** Riley Hospital for Children at Indiana University Health, Indianapolis, Indiana; Richard M. Fairbanks Burn Center, Indianapolis, Indiana; Riley Hospital For Children, Fishers, Indiana; Riley Hospital, Indianapolis, Indiana; Riley Hospital for children, Indianapolis, Indiana

## Abstract

**Introduction:**

Pediatric patients with facial burns and advanced airway needs precipitate acute situations requiring multidisciplinary team member collaboration. Significant facial burns, particularly involving considerable edema or smoke inhalation, may warrant dental or circum-mandibular endotracheal tube (ETT) wiring for stabilization. Guidelines were created, trialed, and revised based on patient outcomes and clinician feedback at a pediatric verified burn center.

**Methods:**

The guideline was created by the burn team in 2019. This standard work was utilized with pediatric burn cases presenting to the burn center. Guideline variances, such as prolonged time from door to ETT wiring, prompted a gap analysis need to improve the process. Through two case review sessions held in 2020, a multidisciplinary team consisting of the burn providers, emergency department providers, nursing, and respiratory therapists revealed process knowledge deficits, unclear role expectations, and supply issues. Literature was reviewed and a myAmeriburn listserv inquiry of current practice was made to seek additional guidance.

**Results:**

Based on feedback from the multidisciplinary group and data gathered from the literature and collegial burn community, action plans and guideline modifications were developed in 2021. The respiratory therapy department developed education for their staff highlighting use of twill tape and taping. The use of ETT suturing was removed from the guidelines.

**Conclusions:**

Multidisciplinary contribution and engagement was necessary to produce, execute, and evaluate the pediatric endotracheal tube wiring guidelines. Through dialogue, patient trial, and constructive feedback, guidelines were amended to produce a smoother team process and better patient experience. The standard work is currently being evaluated in its modified version.

